# Supplementary material for: Shifts in ophthalmic care utilization during the COVID-19 pandemic in the US
Source: Commun Med (Lond). 2023 Dec 14;3:181. doi: 10.1038/s43856-023-00416-4 (PMC10721809; doi:10.1038/s43856-023-00416-4)
Supplement: Supplementary file 1 — Supplementary Information [file 43856_2023_416_MOESM1_ESM.pdf]

Supplementary Information

Table of Contents

Li C, Lum F, Chen E, Collender P, Head J, Khurana R, Cunningham E, Moorthy R, Parke D, McLeod S. *Shifts in ophthalmic care utilization during the COVID-19 pandemic in the US*.

Supplementary Note 1	Model specifications for candidate counterfactual models	Pg. 2
Supplementary Note 2	Adjusting for overdispersion in conditional Poisson GLMs	Pg. 3
Supplementary Note 3	Description and results of sensitivity analyses	Pg. 4
Supplementary Note 4	Construction of diagnosis entities and categories	Pgs. 5-6
Supplementary Figure 1	Hiatus vs. post-hiatus deviations for each diagnosis category	Pg. 7
Supplementary Figure 2	Hiatus vs. post-hiatus deviations for retinal conditions (besides DR and AMD)	Pg. 8
Supplementary Figure 3	Hiatus vs. post-hiatus deviations for corneal and external conditions	Pg. 9
Supplementary Figure 4	Hiatus vs. post-hiatus deviations for oculofacial plastics and orbital conditions	Pg. 10
Supplementary Figure 5	Hiatus vs. post-hiatus deviations for uveitis and ocular inflammation	Pg. 11
Supplementary Figure 6	Hiatus vs. post-hiatus deviations for blindness and vision defects	Pg. 12
Supplementary Figure 7	Heatmap of monthly deviations for all 261 diagnosis entities included for analysis	Pgs. 13-16
Supplementary Table 1	Mapping of diagnosis entities in this study to the ocular emergencies surveyed by Bourges et al.	Pgs. 17-18
Supplementary Table 2	Vision-threatening (VT) and non-vision threatening (NVT) diagnosis entities related to diabetic retinopathy, age-related macular degeneration, and glaucoma	Pg. 19
Supplementary Table 3	Extreme deviations from expectation in the post-hiatus period	Pgs. 20-21
Supplementary Table 4	Diagnosis entities exhibiting partial or sustained recovery in the post-hiatus period	Pgs. 22-23
Supplementary References	References for the Supplementary Information	Pg. 24

Supplementary Note 1. Model specifications for candidate counterfactual models.

To predict the number of monthly patients expected for each diagnosis entity, ten candidate predictive generalized linear models (GLMs) were specified based on various combinations of terms for seasonality and yearly trend:

1. $Y_t \sim \beta_0 + \beta_1 \times year + \sum_{i=1}^{11}[\alpha_i \times I(month_i)]$
2. $Y_t \sim \beta_0 + \beta_1 \times year + \sum_{i=1}^{11}[\alpha_i \times I(month_i)] + \beta_2 sin\frac{2\pi t}{3} + \beta_3 cos\frac{2\pi t}{3}$
3. $Y_t \sim \beta_0 + \beta_1 \times year + \sum_{i=1}^{11}[\alpha_i \times I(month_i)] + \beta_2 sin\frac{2\pi t}{12} + \beta_3 cos\frac{2\pi t}{12}$
4. $Y_t \sim \beta_0 + \beta_1 \times year + \sum_{i=1}^{11}[\alpha_i \times I(month_i)] + \beta_2 sin\frac{2\pi t}{6} + \beta_3 cos\frac{2\pi t}{6}$
5. $Y_t \sim \beta_0 + \beta_1 \times year + \sum_{i=1}^{11}[\alpha_i \times I(month_i)] + \beta_2 sin\frac{2\pi t}{6} + \beta_3 cos\frac{2\pi t}{6} + \beta_4 sin\frac{2\pi t}{12} + \beta_5 cos\frac{2\pi t}{12}$
6. $Y_t \sim \beta_0 + \beta_1 \times year + \sum_{i=1}^{11}[\alpha_i \times I(month_i)] + \beta_2 sin\frac{2\pi t}{6} + \beta_3 cos\frac{2\pi t}{6} + \beta_4 sin\frac{2\pi t}{3} + \beta_5 cos\frac{2\pi t}{3}$
7. $Y_t \sim \beta_0 + \beta_1 \times year + \sum_{i=1}^{11}[\alpha_i \times I(month_i)] + \beta_2 sin\frac{2\pi t}{12} + \beta_3 cos\frac{2\pi t}{12} + \beta_4 sin\frac{2\pi t}{3} + \beta_5 cos\frac{2\pi t}{3}$
8. $Y_t \sim \beta_0 + \beta_1 \times year + \sum_{i=1}^{11}[\alpha_i \times I(month_i)] + \beta_2 sin\frac{2\pi t}{3} + \beta_3 cos\frac{2\pi t}{3} + \beta_4 sin\frac{2\pi t}{6} + \beta_5 cos\frac{2\pi t}{6} + \beta_6 sin\frac{2\pi t}{12} + \beta_7 cos\frac{2\pi t}{12}$
9. $Y_t \sim \beta_0 + \beta_1 \times year + \beta_2 sin\frac{2\pi t}{3} + \beta_3 cos\frac{2\pi t}{3} + \beta_4 sin\frac{2\pi t}{6} + \beta_5 cos\frac{2\pi t}{6} + \beta_6 sin\frac{2\pi t}{12} + \beta_7 cos\frac{2\pi t}{12}$
10. $Y_t \sim \beta_0 + \sum_{i=1}^{11}[\alpha_i \times I(month_i)] + \beta_1 sin\frac{2\pi t}{3} + \beta_2 cos\frac{2\pi t}{3} + \beta_3 sin\frac{2\pi t}{6} + \beta_4 cos\frac{2\pi t}{6} + \beta_5 sin\frac{2\pi t}{12} + \beta_6 cos\frac{2\pi t}{12}$

where:

Y_t = number of patients reported with the diagnosis entity in month t since January 2017, for $t \in (1, 2, \ldots, 36)$

$year$ = continuous term on year

$I(month_i)$ = indicator of calendar month

All candidate counterfactual models initially assumed conditionally Poisson-distributed outcomes.

Supplementary Note 2. Adjusting for overdispersion in Poisson GLMs.

An assumption of the Poisson generalized linear model (GLM) is that the variance of the data equals the mean; but in reality, the data often exhibit greater variability than its mean.[1] This phenomenon is known as overdispersion, and is commonly accounted for using one of two models closely related to the Poisson GLM: the negative binomial (NB) or the quasi-Poisson (QP) regression model. For the NB model, variance is quadratically related to the mean; whereas for the QP model, the variance is a linear function of the mean.[2]

Before undertaking steps to decide between the NB or QP model, we first conducted a two-sided dispersion test[3] to detect whether the data were overdispersed with statistical significance (alpha = 0.05). For diagnosis entities that were not overdispersed **(Supplementary Data 2)**, we retained the mean predictions and standard error estimates from the initial Poisson GLM to establish the counterfactual expectations, and 95% prediction intervals associated with them.

For diagnosis entities that were overdispersed **(Supplementary Data 2)**, to decide whether a NB or QP model would be more appropriate to adjust for overdispersion, we examined whether the residuals and their predictions varied quadratically or linearly. That is, following the recommendations of Ver Hoef and Boveng,[2] we regressed the squared predicted values and the predicted values against the residuals. If the regression with the quadratic term had a lower Akaike Information Criterion (AIC) than the regression with the linear term, we used a NB model; otherwise, we used a QP model to adjust for overdispersion.

Supplementary Note 3. Description and results of sensitivity analyses.

We performed sensitivity analyses to demonstrate no major changes in primary outcomes (the average deviations across all diagnosis entities at various time points) across different counterfactual model performance thresholds (minimum RMSPE cutoffs) or sets of inclusion criteria applied to determine which records or patients were eligible for inclusion in the study.

Across various combinations of inclusion criteria, we also found that requiring that (a) patients have a physician encounter (defined as having a valid procedure, ocular exam result, or visit record on the same date as that of the diagnosis documentation) documented on the same date of diagnosis documentation, *and* (b) excluding practices that did not contribute data for each month in the global study period, yielded the lowest counterfactual model errors. The final sets of inclusion and model performance criteria used for this study are highlighted in yellow.

Inclusion Criteria		Model Performance Criteria (maximum allowable error, i.e., RMSPE)	Mean (SD) Deviations Across All Diagnosis Entities		Final Number of Diagnosis Entities Included
(a): same-day visit	(b): complete data for all practices		April 2020	Post-Hiatus (June 2020 - December 2021)	
FALSE	FALSE	12.5%	-0.676 (0.128)	-0.148 (0.100)	249
FALSE	FALSE	10.0%	-0.680 (0.123)	-0.152 (0.075)	218
TRUE	FALSE	12.5%	-0.682 (0.126)	-0.146 (0.098)	248
TRUE	FALSE	10.0%	-0.689 (0.122)	-0.149 (0.073)	218
TRUE	TRUE	12.5%	-0.665 (0.138)	-0.128 (0.088)	261
TRUE	TRUE	10.0%	-0.670 (0.136)	-0.133 (0.068)	224

Inclusion Criteria		Mean (SD) Counterfactual Model Error (RMSPE)
(a): same-day visit	(b): complete data for all practices	
FALSE	FALSE	10.8% (8.0%)
TRUE	FALSE	10.7% (8.0%)
TRUE	TRUE	10.3% (7.3%)

Supplementary Note 4. Construction of diagnosis entities and categories.

To construct an inventory of clinically meaningful diagnosis entities to study, we adapted and refined existing categorizations of *International Classification of Diseases, 10th Revision, Clinical Modification* (ICD-10-CM) codes organized by the US Agency for Healthcare Research and Quality Clinical Classifications Software Refined (CCSR) database, version 2020.2 (https://hcup-us.ahrq.gov/toolssoftware/ccsr/ccsr_archive.jsp#ccsr). The full CCSR database contains groupings of more than 70,000 ICD-10-CM diagnosis codes into over 530 clinically meaningful categories; but for this study, we only considered ICD-10-CM codes that describe eye diseases and conditions by first examining the diagnosis codes attributed to the following 12 CCSR categories:

EYE001	Cornea and external disease
EYE002	Cataract and other lens disorders
EYE003	Glaucoma
EYE004	Uveitis and ocular inflammation
EYE005	Retinal and vitreous conditions
EYE006	Neuro-ophthalmology
EYE007	Strabismus
EYE008	Oculofacial plastics and orbital conditions
EYE009	Refractive error
EYE010	Blindness and vision defects
EYE011	Postprocedural or postoperative eye complication
EYE012	Other specified eye disorders

These CCSR categories are identical to 12 (out of 13 total) diagnosis categories used in this study; however, we created an additional diagnosis category of "ocular globe injuries/intraocular foreign bodies" (OGI/IOFB) to capture some eye injury diagnosis codes that were originally assigned to different (non-ophthalmic) categories of the CCSR than the ones listed above, as well as diagnosis codes that indicate the presence of a foreign body in the eye, which were previously assigned by the CCSR into the "Other specified eye disorders" (EYE012) category.

Because this study relied on a finer level of informational granularity about diagnoses than that provided by the broader categorizations of the CCSR, we further grouped the ICD-10-CM codes within each diagnosis category into clinically relevant diagnosis entities. The development and refinement of these entities were undertaken in consultation with board-certified ophthalmologists and clinical experts, and were guided by key principles and considerations:

- *Clinical Relevance*. We aimed to construct diagnosis entities that provided adequate granularity (e.g., allowing differentiation between less and more severe or urgent forms of the same disease) while minimizing redundancy (e.g., avoiding separate groupings for numerous types of clinically similar conditions). For instance, diabetic retinopathy (DR) is a disease that has many subtypes, represented by a wide array of ICD-10 codes that

encode various stages, manifestations, and etiologies. However, the most clinically relevant distinction that would influence the treatment or prognosis of DR is whether diabetic macular edema (DME) is present. Thus, instead of constructing a single coarse entity of “diabetic retinopathy” or several different diagnosis entities for all DR subtypes, we created four diagnosis entities that were most clinically relevant: non-proliferative DR without DME, non-proliferative DR with DME, proliferative DR without DME, proliferative DR with DME.

- *Sufficient Utilization Level.* Each diagnosis entity was required to have above single-digit patient counts for each month of our study period from January 2017 to December 2021, to avoid including conditions that were too rare to the point of introducing instability or noise into our analysis.

- *Mutually Exclusive Assignment to Diagnosis Categories.* To provide a precise framework for analysis, each ICD-10-CM code was mapped to exactly one diagnosis entity, and each diagnosis entity was mapped to exactly one diagnosis category. Unlike v2020.2 of the CCSR, which allows for individual ICD-10-CM codes to be mapped to more than one category, we adopted a mutually exclusive categorization scheme to ensure clarity in the interpretation of data by avoiding overlapping categorizations that can make comparisons across categories less meaningful. If an ICD-10-CM code was originally assigned to more than one category in the CCSR, its assignment to a single category for this study was made based on a consideration of its primary clinical significance.

Supplementary Figure 1. Hiatus vs. post-hiatus deviations for each diagnosis category.

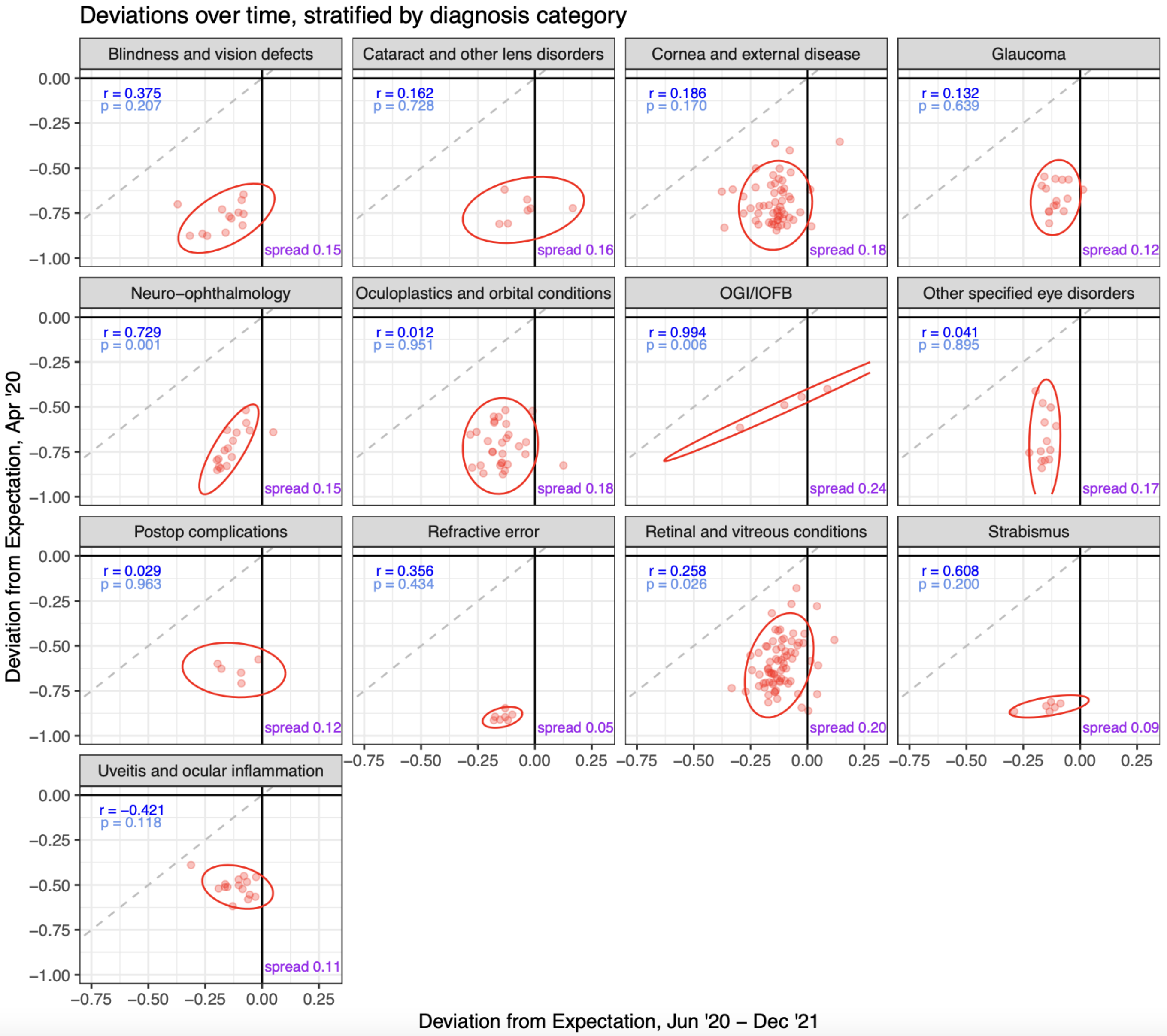

Deviations during the nadir of the hiatus period (April 2020) are plotted against deviations in the post-hiatus period (June 2020 - December 2021), for all diagnosis entities (red points) that belong to each diagnosis category. 95% normal data ellipses (red ovals) represent estimated probability contours expected to contain 95% of all plotted diagnosis entities within each category, and a line of equality (dashed gray line) represents no change in deviations over time (i.e., deviations during April 2020 are equal to the average post-hiatus deviations). For the distribution of points within each category, a Pearson's product moment correlation coefficient (r) is reported in the upper left corner along with its p-value, as well as the average of all pairwise Euclidean distances between points in the distribution (*spread*; lower right corner). *Abbreviations*: OGI/IOFB = ocular globe injury/intraocular foreign body.

Supplementary Figure 2. Hiatus vs. post-hiatus deviations for retinal conditions (besides DR and AMD).

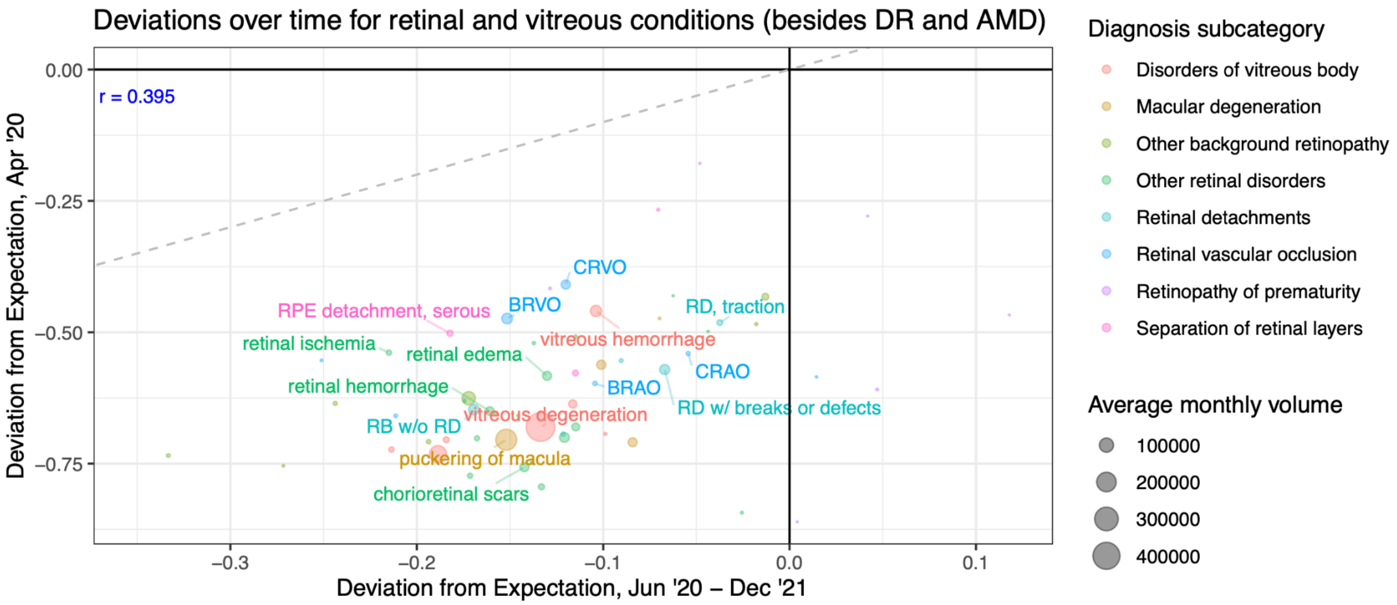

Deviations during the nadir of the hiatus period (April 2020) are plotted against deviations in the post-hiatus period (June 2020 - December 2021), for diagnosis entities that correspond to retinal and vitreous conditions aside from the diagnoses of diabetic retinopathy (DR) and age-related macular degeneration (AMD) that are plotted in **Figure 3**. The size of each point corresponds to the average number of monthly patients for the diagnosis entity. Points were selectively labeled based on relevance (e.g., we excluded labels of diagnosis entities whose names contain key words like *other* or *unspecified*). A Pearson's product moment correlation coefficient (r) is reported for the distribution of points in each plot (blue text; upper left corner), and a line of equality (dashed gray line) represents no change in deviations over time. *Abbreviations*: CRVO = central retinal vein occlusion; BRVO = branch retinal vein occlusion; RD = retinal detachment; RPE = retinal pigment epithelium; RB = retinal break.

Supplementary Figure 3. Hiatus vs. post-hiatus deviations for corneal and external conditions.

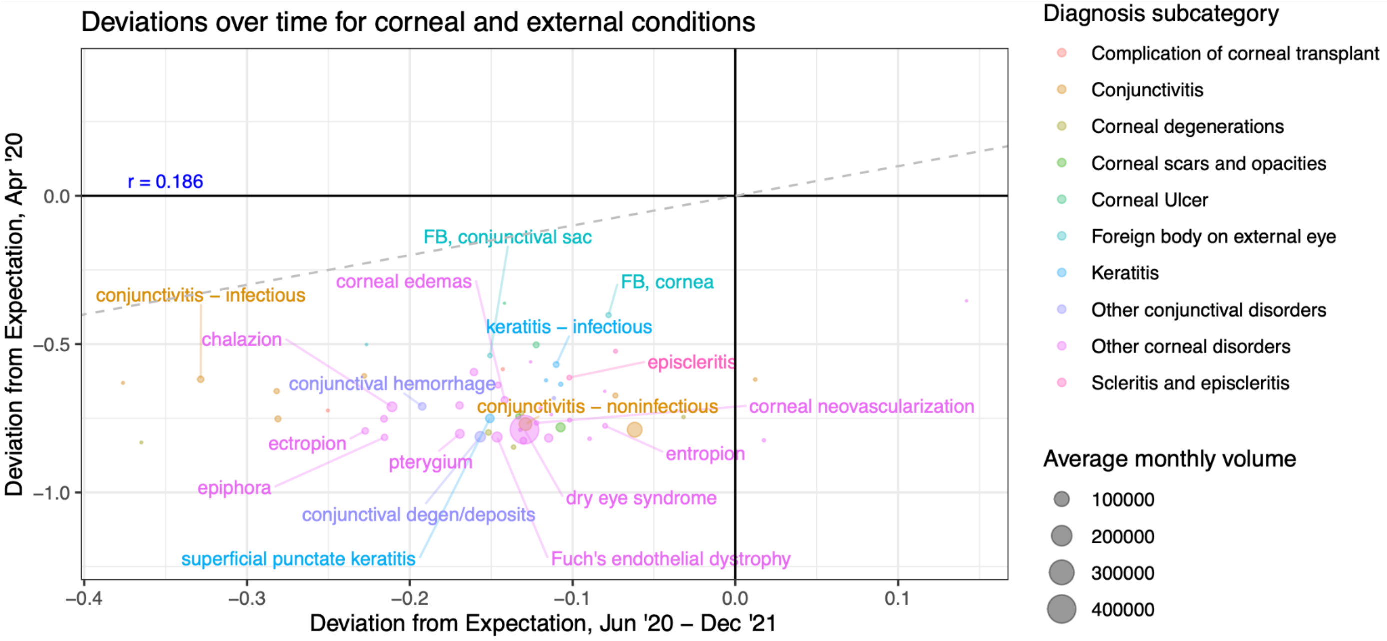

Deviations during the nadir of the hiatus period (April 2020) are plotted against deviations in the post-hiatus period (June 2020 - December 2021), for diagnosis entities that correspond to corneal and external conditions. The size of each point corresponds to the average number of monthly patients for the diagnosis entity. Points were selectively labeled based on relevance (e.g., we excluded labels of diagnosis entities whose names contain key words like *other* or *unspecified*). A Pearson's product moment correlation coefficient (r) is reported for the distribution of points in each plot (blue text; upper left corner), and a line of equality (dashed gray line) represents no change in deviations over time. *Abbreviations*: FB = foreign body.

Supplementary Figure 4. Hiatus vs. post-hiatus deviations for oculofacial plastics and orbital conditions.

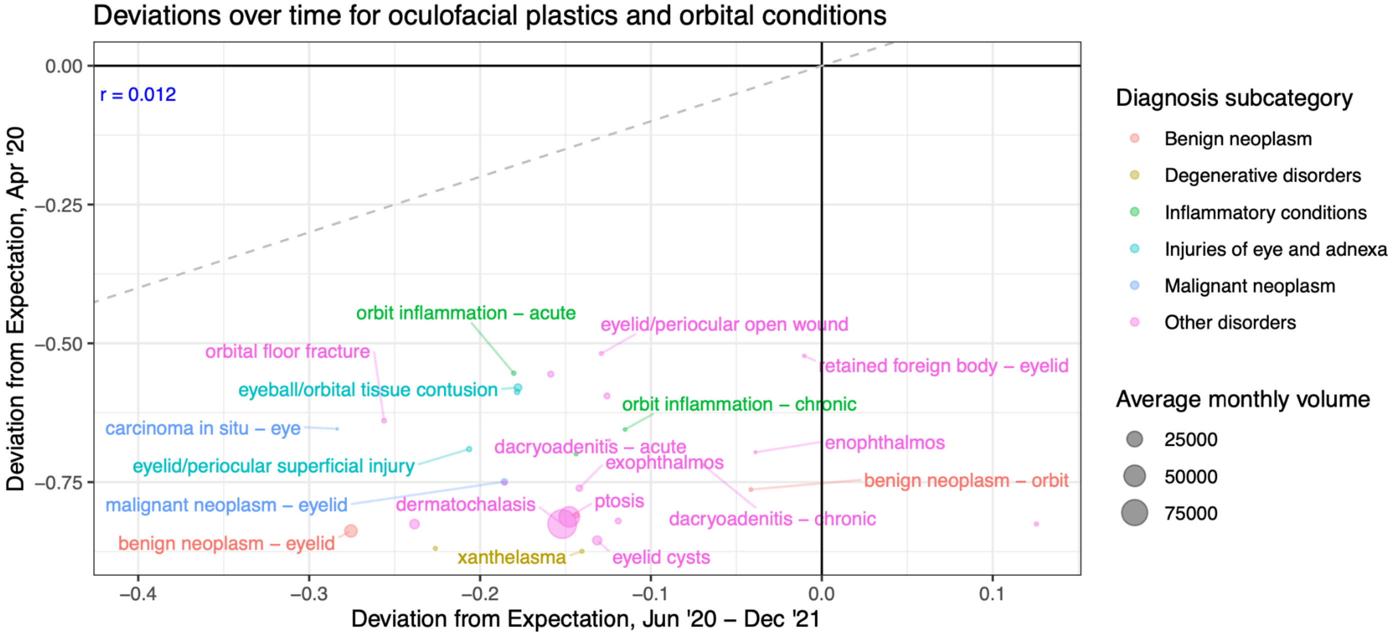

Deviations during the nadir of the hiatus period (April 2020) are plotted against deviations in the post-hiatus period (June 2020 - December 2021), for diagnosis entities that correspond to oculofacial plastics and orbital conditions. The size of each point corresponds to the average number of monthly patients for the diagnosis entity. Points were selectively labeled based on relevance (e.g., we excluded labels of diagnosis entities whose names contain key words like *other* or *unspecified*). A Pearson's product moment correlation coefficient (r) is reported for the distribution of points in each plot (blue text; upper left corner), and a line of equality (dashed gray line) represents no change in deviations over time.

Supplementary Figure 5. Hiatus vs. post-hiatus deviations for uveitis and ocular inflammation.

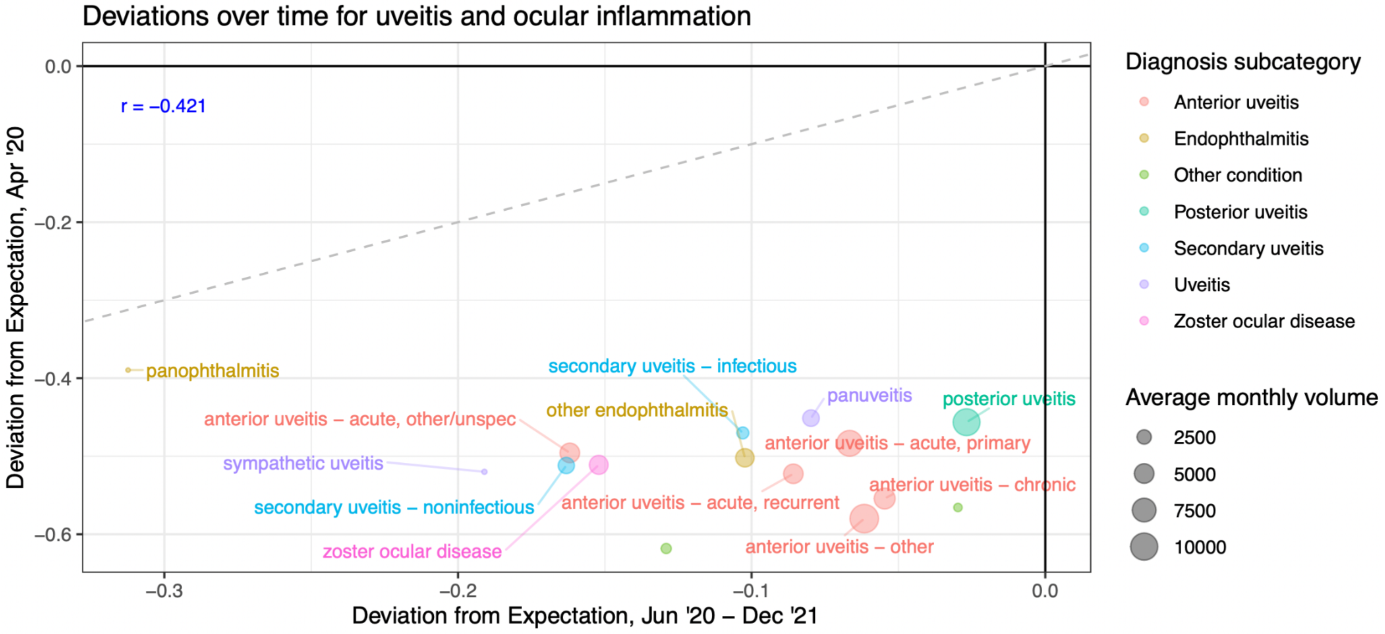

Deviations during the nadir of the hiatus period (April 2020) are plotted against deviations in the post-hiatus period (June 2020 - December 2021), for diagnosis entities that correspond to diagnoses of uveitis and ocular inflammation. The size of each point corresponds to the average number of monthly patients for the diagnosis entity. Points were selectively labeled based on relevance (e.g., we excluded labels of diagnosis entities whose names contain key words like *other* or *unspecified*). A Pearson's product moment correlation coefficient (r) is reported for the distribution of points in each plot (blue text; upper left corner), and a line of equality (dashed gray line) represents no change in deviations over time.

Supplementary Figure 6. Hiatus vs. post-hiatus deviations for blindness and vision defects.

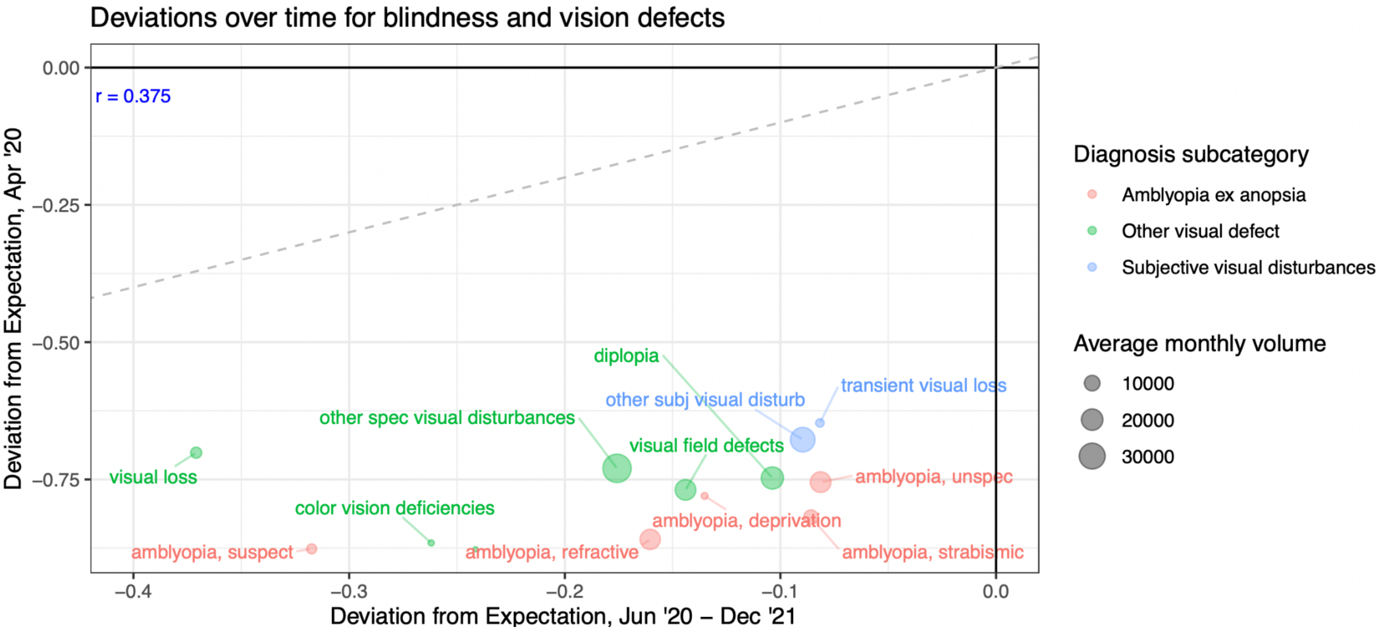

Deviations during the nadir of the hiatus period (April 2020) are plotted against deviations in the post-hiatus period (June 2020 - December 2021), for diagnosis entities that correspond to diagnoses of blindness and vision defects. The size of each point corresponds to the average number of monthly patients for the diagnosis entity. A Pearson's product moment correlation coefficient (r) is reported for the distribution of points in each plot (blue text; upper left corner), and a line of equality (dashed gray line) represents no change in deviations over time. *Abbreviations*: subj = subjective; spec = specified; unspec = unspecified.

Supplementary Figure 7. Heatmap of monthly deviations for all 261 diagnosis entities included for analysis.

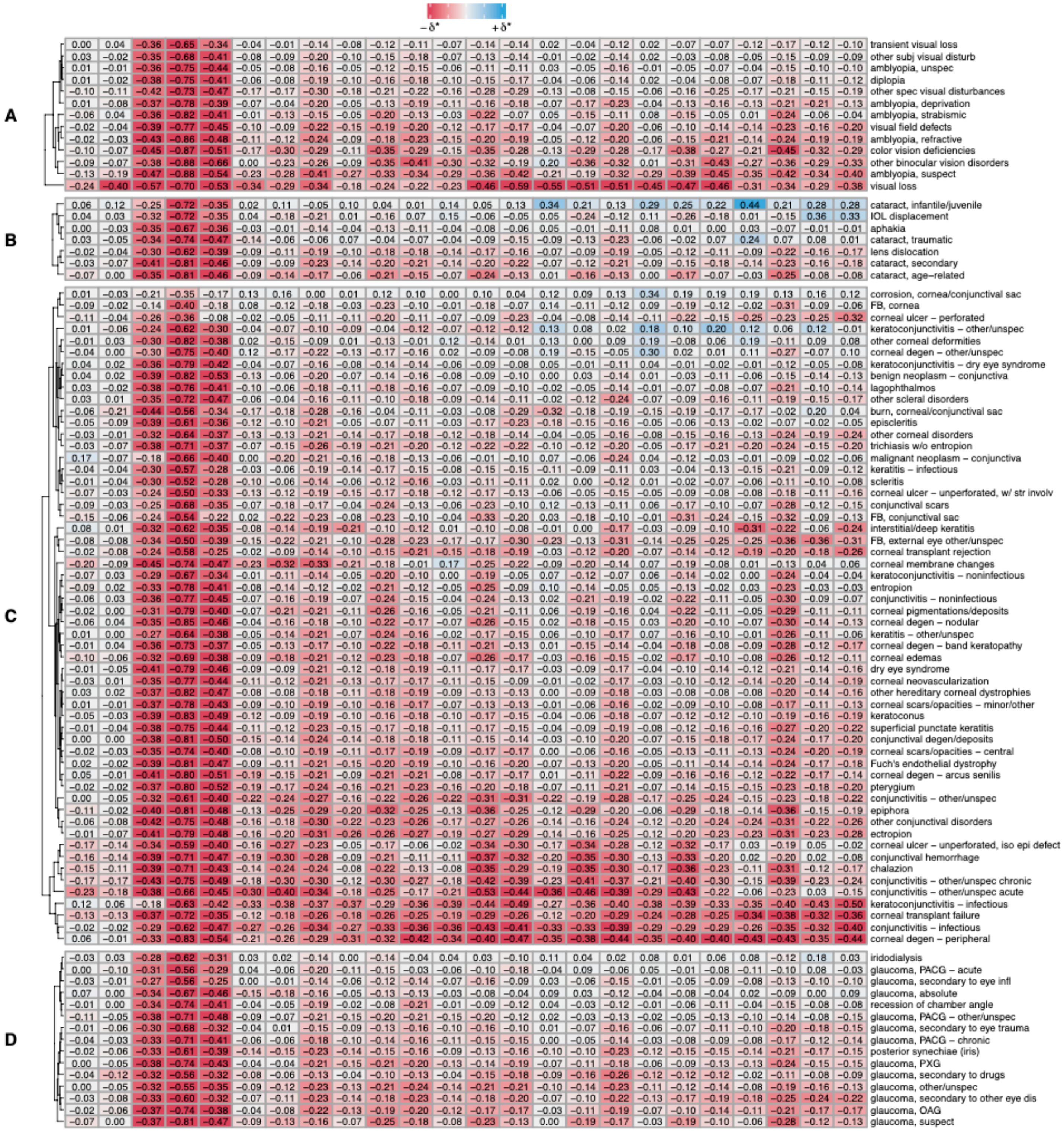

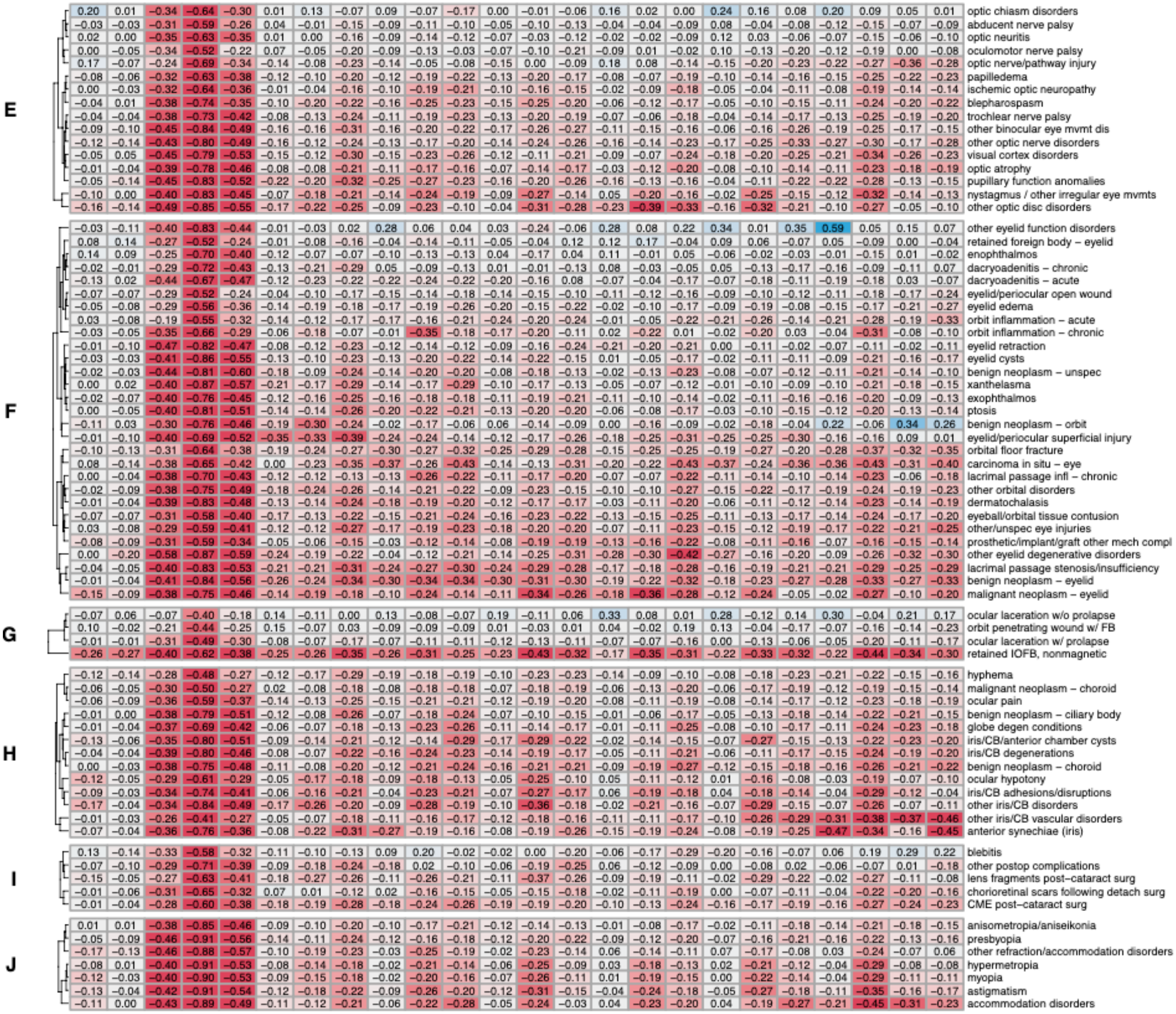
E
optic chiasm disorders
abducent nerve palsy
optic neuritis
oculomotor nerve palsy
optic nerve/pathway injury
papilledema
ischemic optic neuropathy
blepharospasm
trochlear nerve palsy
other binocular eye mvmt dis
other optic nerve disorders
visual cortex disorders
optic atrophy
pupillary function anomalies
nystagmus / other irregular eye mvmts
other optic disc disorders
F
other eyelid function disorders
retained foreign body – eyelid
enophthalmos
dacryoadenitis – chronic
dacryoadenitis – acute
eyelid/periocular open wound
eyelid edema
orbit inflammation – acute
orbit inflammation – chronic
eyelid retraction
eyelid cysts
benign neoplasm – unspec
xanthelasma
exophthalmos
ptosis
benign neoplasm – orbit
eyelid/periocular superficial injury
orbital floor fracture
carcinoma in situ – eye
lacrimal passage infl – chronic
other orbital disorders
dermatochalasis
eyeball/orbital tissue contusion
other/unspec eye injuries
prosthetic/implant/graft other mech compl
other eyelid degenerative disorders
lacrimal passage stenosis/insufficiency
benign neoplasm – eyelid
malignant neoplasm – eyelid
G
ocular laceration w/o prolapse
orbit penetrating wound w/ FB
ocular laceration w/ prolapse
retained IOFB, nonmagnetic
H
hyphema
malignant neoplasm – choroid
ocular pain
benign neoplasm – ciliary body
globe degen conditions
iris/CB/anterior chamber cysts
iris/CB degenerations
benign neoplasm – choroid
ocular hypotony
iris/CB adhesions/disruptions
other iris/CB disorders
other iris/CB vascular disorders
anterior synechiae (iris)
I
blebitis
other postop complications
lens fragments post-cataract surg
chorioretinal scars following detach surg
CME post-cataract surg
J
anisometropia/aniseikonia
presbyopia
other refraction/accommodation disorders
hypermetropia
myopia
astigmatism
accommodation disorders

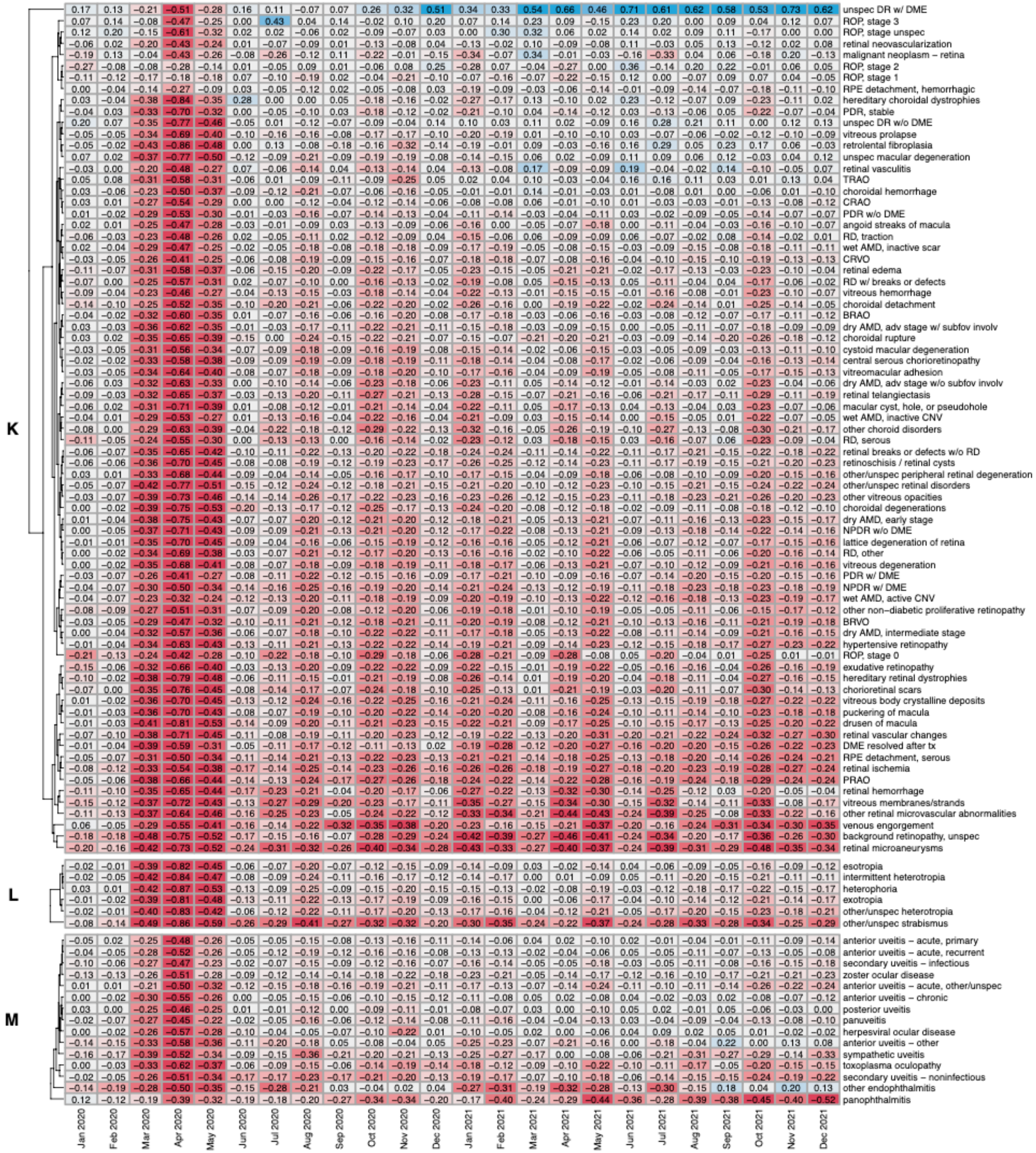
K
L
M
unspec DR w/ DME
ROP, stage 3
ROP, stage unspec
retinal neovascularization
malignant neoplasm – retina
ROP, stage 2
ROP, stage 1
RPE detachment, hemorrhagic
hereditary choroidal dystrophies
PDR, stable
unspec DR w/o DME
vitreous prolapse
retrolental fibroplasia
unspec macular degeneration
retinal vasculitis
TRAO
choroidal hemorrhage
CRAO
PDR w/o DME
angoid streaks of macula
RD, traction
wet AMD, inactive scar
CRVO
retinal edema
RD w/ breaks or defects
vitreous hemorrhage
choroidal detachment
BRAO
dry AMD, adv stage w/ subfov involv
choroidal rupture
cystoid macular degeneration
central serous chorioretinopathy
vitreomacular adhesion
dry AMD, adv stage w/o subfov involv
retinal telangiectasis
macular cyst, hole, or pseudohole
wet AMD, inactive CNV
other choroid disorders
RD, serous
retinal breaks or defects w/o RD
retinoschisis / retinal cysts
other/unspec peripheral retinal degeneration
other/unspec retinal disorders
other vitreous opacities
choroidal degenerations
dry AMD, early stage
NPDR w/o DME
lattice degeneration of retina
RD, other
vitreous degeneration
PDR w/ DME
NPDR w/ DME
wet AMD, active CNV
other non–diabetic proliferative retinopathy
BRVO
dry AMD, intermediate stage
hypertensive retinopathy
ROP, stage 0
exudative retinopathy
hereditary retinal dystrophies
chorioretinal scars
vitreous body crystalline deposits
puckering of macula
drusen of macula
retinal vascular changes
DME resolved after tx
RPE detachment, serous
retinal ischemia
PRAO
retinal hemorrhage
vitreous membranes/strands
other retinal microvascular abnormalities
venous engorgement
background retinopathy, unspec
retinal microaneurysms
esotropia
intermittent heterotropia
heterophoria
exotropia
other/unspec heterotropia
other/unspec strabismus
anterior uveitis – acute, primary
anterior uveitis – acute, recurrent
secondary uveitis – infectious
zoster ocular disease
anterior uveitis – acute, other/unspec
anterior uveitis – chronic
posterior uveitis
panuveitis
herpesviral ocular disease
anterior uveitis – other
sympathetic uveitis
toxoplasma oculopathy
secondary uveitis – noninfectious
other endophthalmitis
panophthalmitis
Jan 2020
Feb 2020
Mar 2020
Apr 2020
May 2020
Jun 2020
Jul 2020
Aug 2020
Sep 2020
Oct 2020
Nov 2020
Dec 2020
Jan 2021
Feb 2021
Mar 2021
Apr 2021
May 2021
Jun 2021
Jul 2021
Aug 2021
Sep 2021
Oct 2021
Nov 2021
Dec 2021

Cluster heatmap of deviations for each month of the pandemic study period for all 261 diagnosis entities, partitioned by 13 diagnosis categories. A diverging color scale in the heatmap is used to illustrate the direction, magnitude, and statistical significance of each monthly deviation, with the darkness of each cell a function of the product between the magnitude of the deviation and the negative log of its adjusted p-value. Clusters of diagnosis entities within each partition were identified using a hierarchical clustering algorithm applied to the rows of the heatmap (i.e., to the vectors of monthly deviations), and are represented by the dendrograms to the left of the heatmap, and the subsequent ordering of the diagnosis entities within each partition.

Legend for partitions:

Code	Diagnosis Category
**A**	Blindness and vision defects
**B**	Cataract and other lens disorders
**C**	Cornea and external disease
**D**	Glaucoma
**E**	Neuro-ophthalmology
**F**	Oculofacial plastics and orbital conditions
**G**	OGI/IOFB
**H**	Other specified eye disorders
**I**	Postprocedural or postoperative eye complication
**J**	Refractive error
**K**	Retinal and vitreous conditions
**L**	Strabismus
**M**	Uveitis and ocular inflammation

Abbreviations: Where necessary, please refer to the codebook in **Supplementary Data 1** for the full diagnosis names of the 261 diagnosis entities visualized in this heatmap.

Supplementary Table 1. Mapping of diagnosis entities in this study to the ocular emergencies surveyed by Bourges et al.

Adapted from Table 2 of *Bourges et al*.[4]

Category	Subcategory	Item	Severity	Diagnosis Entity Name
anterior segment	conjunctivitis	noninfectious	1	conjunctivitis - noninfectious
anterior segment	keratitis	superficial punctate keratitis	1	superficial punctate keratitis
anterior segment	pterygia	inflamed pterygia	1	pterygium
anterior segment	conjunctivitis	viral conjunctivitis	2	conjunctivitis - infectious
anterior segment	scleral/episcleral inflammation	episcleritis (regardless of the cause)	2	episcleritis
anterior segment	corneal ulcer (not infected)	unperforated, isolated epithelial defect	2	corneal ulcer - unperforated, iso epi defect
anterior segment	conjunctivitis	bacterial conjunctivitis	2	conjunctivitis - infectious
anterior segment	anterior acute uveitis	iterative (regardless of the cause)	3	anterior uveitis - acute, recurrent
anterior segment	scleral/episcleral inflammation	scleritis (regardless of the cause)	3	scleritis
anterior segment	corneal ulcer (not infected)	unperforated, with stromal involvement	3	corneal ulcer - unperforated, w/ str involv
anterior segment	keratitis	infectious keratitis	3	keratitis - infectious
anterior segment	ocular surface burn	<9 clock hours of limbus and <75% of conjunctiva	3	burn, corneal/conjunctival sac
anterior segment	glaucoma	acute angle-closure glaucoma	4	glaucoma, PACG - acute
anterior segment	corneal ulcer (not infected)	perforating ulcer	5	corneal ulcer - perforated
posterior segment	nonspecific visual symptoms	vitreous floaters	1	other vitreous opacities
posterior segment	macula	macular edema	3	retinal edema
posterior segment	retinal peripheral tear	no detached edges, no RD associated	3	retinal breaks or defects w/o RD
posterior segment	macula	macular hole	3	macular cyst, hole, or pseudohole
posterior segment	retinal vein occlusion	branch (BRVO)	3	BRVO
posterior segment	retinal peripheral tear	detached edges, no RD associated	3	retinal breaks or defects w/o RD
posterior segment	posterior segment inflammation	retinal vasculitis	4	retinal vasculitis
posterior segment	retinal artery occlusion	branch (BRAO)	4	BRAO
posterior segment	retinal vein occlusion	central (CRVO)	4	CRVO
posterior segment	ischemic optic neuropathy	isolated	4	ischemic optic neuropathy
posterior segment	retinal artery occlusion	central (CRAO)	5	CRAO

Category	Subcategory	Item	Severity	Diagnosis Entity Name
traumatisms	conjunctiva	subconjunctival hemorrhage	1	conjunctival hemorrhage
traumatisms	other injuries	eyelid skin injury	2	eyelid/periocular superficial injury
traumatisms	blunt injuries with impaired vision	hyphema (isolated)	3	hyphema
traumatisms	other injuries	eyelid-margin injury	3	eyelid/periocular open wound
traumatisms	blunt injuries with impaired vision	iridodialysis (avulsion of the iris root)	3	iridodialysis
traumatisms	blunt injuries with impaired vision	choroidal rupture	4	choroidal rupture
traumatisms	blunt injuries with impaired vision	lens dislocation	4	lens dislocation
traumatisms	corneal injury	perforating and leaking laceration	5	ocular laceration w/ prolapse
traumatisms	penetrating eye injury	with intraocular foreign body, eye not collapsed	5	orbit penetrating wound w/ FB
traumatisms	blunt injuries with impaired vision	scleral rupture	5	ocular laceration w/ prolapse
traumatisms	other injuries	optic nerve injury	5	optic nerve/pathway injury
traumatisms	penetrating eye injury	with intraocular foreign body, eye collapsed	5	orbit penetrating wound w/ FB
post-op complications	keratoplasty	acute rejection	4	corneal transplant rejection
post-op complications	infection following surgery	blebitis	5	blebitis
post-op complications	infection following surgery	panophthalmitis	5	panophthalmitis

Supplementary Table 2. Vision-threatening (VT) and non-vision threatening (NVT) diagnosis entities related to diabetic retinopathy, age-related macular degeneration, and glaucoma.

Diagnosis Entity	Category	Classification
PDR w/ DME	DR	VT
PDR w/o DME	DR	VT
unspec DR w/ DME	DR	VT
NPDR w/ DME	DR	VT
PDR, stable	DR	NVT
DME resolved after tx	DR	NVT
unspec DR w/o DME	DR	NVT
NPDR w/o DME	DR	NVT
dry AMD, adv stage w/o subfov involv	AMD	VT
dry AMD, adv stage w/ subfov involv	AMD	VT
wet AMD, active CNV	AMD	VT
wet AMD, inactive CNV	AMD	VT
wet AMD, inactive scar	AMD	VT
unspec macular degeneration	AMD	NVT
drusen of macula	AMD	NVT
dry AMD, early stage	AMD	NVT
dry AMD, intermediate stage	AMD	NVT
glaucoma, absolute	Glaucoma	VT
glaucoma, PACG - acute	Glaucoma	VT
glaucoma, PACG - chronic	Glaucoma	VT
glaucoma, PACG - other/unspec	Glaucoma	VT
glaucoma, secondary to drugs	Glaucoma	VT
glaucoma, secondary to eye infl	Glaucoma	VT
glaucoma, secondary to eye trauma	Glaucoma	VT
glaucoma, secondary to other eye dis	Glaucoma	VT
glaucoma, OAG	Glaucoma	NVT
glaucoma, PXG	Glaucoma	NVT
glaucoma, suspect	Glaucoma	NVT

Supplementary Table 3. Extreme deviations from expectation in the post-hiatus period.

A

Entity Name	Post–hiatus Mean Deviation	95% CI	p–value	Recovery Month	Recovery Type	RMSPE
keratoconjunctivitis – infectious	−0.376	(−0.405,−0.346)	< 0.001	No recovery	N/A	0.120
visual loss	−0.371	(−0.395,−0.346)	< 0.001	No recovery	N/A	0.115
corneal degen – peripheral	−0.365	(−0.388,−0.341)	< 0.001	No recovery	N/A	0.090
retinal microaneurysms	−0.333	(−0.353,−0.313)	< 0.001	No recovery	N/A	0.076
conjunctivitis – infectious	−0.328	(−0.35,−0.306)	< 0.001	No recovery	N/A	0.076
amblyopia, suspect	−0.317	(−0.347,−0.287)	< 0.001	No recovery	N/A	0.097
panophthalmitis	−0.312	(−0.342,−0.281)	< 0.001	No recovery	N/A	0.078
retained IOFB, nonmagnetic	−0.297	(−0.318,−0.275)	< 0.001	No recovery	N/A	0.056
other/unspec strabismus	−0.290	(−0.309,−0.27)	< 0.001	No recovery	N/A	0.068
carcinoma in situ – eye	−0.284	(−0.32,−0.245)	< 0.001	No recovery	N/A	0.113
conjunctivitis – other/unspec acute	−0.282	(−0.303,−0.26)	< 0.001	No recovery	N/A	0.065
conjunctivitis – other/unspec chronic	−0.281	(−0.308,−0.253)	< 0.001	No recovery	N/A	0.092
benign neoplasm – eyelid	−0.276	(−0.3,−0.25)	< 0.001	No recovery	N/A	0.088
background retinopathy, unspec	−0.272	(−0.29,−0.252)	< 0.001	No recovery	N/A	0.064
color vision deficiencies	−0.262	(−0.291,−0.232)	< 0.001	No recovery	N/A	0.090
orbital floor fracture	−0.256	(−0.291,−0.22)	< 0.001	No recovery	N/A	0.114
venous engorgement	−0.251	(−0.267,−0.235)	< 0.001	No recovery	N/A	0.065
corneal transplant failure	−0.250	(−0.263,−0.238)	< 0.001	No recovery	N/A	0.047
other retinal microvascular abnormalities	−0.244	(−0.262,−0.225)	< 0.001	No recovery	N/A	0.070
other binocular vision disorders	−0.241	(−0.275,−0.206)	< 0.001	No recovery	N/A	0.123
lacrimal passage stenosis/insufficiency	−0.238	(−0.261,−0.215)	< 0.001	No recovery	N/A	0.085
conjunctivitis – other/unspec	−0.228	(−0.25,−0.206)	< 0.001	No recovery	N/A	0.054
FB, external eye other/unspec	−0.227	(−0.256,−0.196)	< 0.001	No recovery	N/A	0.121
ectropion	−0.227	(−0.249,−0.205)	< 0.001	No recovery	N/A	0.059
other eyelid degenerative disorders	−0.226	(−0.256,−0.195)	< 0.001	No recovery	N/A	0.123
anterior synechiae (iris)	−0.224	(−0.247,−0.2)	< 0.001	No recovery	N/A	0.081
other conjunctival disorders	−0.216	(−0.238,−0.193)	< 0.001	No recovery	N/A	0.076
epiphora	−0.216	(−0.239,−0.191)	< 0.001	No recovery	N/A	0.063
retinal ischemia	−0.215	(−0.229,−0.201)	< 0.001	No recovery	N/A	0.051
vitreous membranes/strands	−0.214	(−0.232,−0.194)	< 0.001	No recovery	N/A	0.047
PRAO	−0.211	(−0.226,−0.196)	< 0.001	No recovery	N/A	0.050
chalazion	−0.211	(−0.23,−0.192)	< 0.001	No recovery	N/A	0.068
eyelid/periocular superficial injury	−0.206	(−0.24,−0.172)	< 0.001	Nov 2020	Partial	0.072

B

Entity Name	Post–hiatus Mean Deviation	95% CI	p–value	Recovery Month	Recovery Type	RMSPE
unspec DR w/ DME	0.459	(0.373,0.552)	< 0.001	Jun 2020	Full	0.102
cataract, infantile/juvenile	0.167	(0.122,0.213)	< 0.001	Jun 2020	Full	0.114
corrosion, cornea/conjunctival sac	0.142	(0.082,0.205)	< 0.001	Jun 2020	Full	0.115
other eyelid function disorders	0.125	(0.065,0.189)	< 0.001	Jun 2020	Full	0.102
ROP, stage 3	0.118	(0.064,0.175)	< 0.001	Jun 2020	Full	0.082
ocular laceration w/o prolapse	0.088	(0.037,0.141)	< 0.001	Jun 2020	Full	0.120
optic chiasm disorders	0.049	(0.017,0.082)	0.00144	Jun 2020	Partial	0.116
ROP, stage unspec	0.047	(−0.004,0.101)	0.06543	Jun 2020	Full	0.108
unspec DR w/o DME	0.043	(−0.005,0.094)	0.06818	Jun 2020	Full	0.108
ROP, stage 2	0.042	(−0.019,0.108)	0.17133	Jun 2020	Full	0.080
other corneal deformities	0.018	(−0.011,0.047)	0.21815	Nov 2020	Full	0.064
TRAO	0.014	(−0.015,0.045)	0.33402	Jun 2020	Partial	0.087
iridodialysis	0.013	(−0.016,0.042)	0.37551	Jun 2020	Full	0.084
keratoconjunctivitis – other/unspec	0.012	(−0.001,0.026)	0.0619	Mar 2021	Full	0.108
retrolental fibroplasia	0.004	(−0.047,0.059)	0.87671	Jun 2020	Partial	0.093

Diagnosis Category

- Blind
- Cataract
- Cornea/External
- Glaucoma
- Neuro
- Oculoplastics
- OGI
- Other
- Retina/Vitreous
- Strab
- Uveitis

Diagnosis entities that (A) exhibited the largest suppressions in care utilization during the post-hiatus period (i.e., with a mean deviation ≤ -20%), and (B) met or exceeded counterfactual expectations in the post-hiatus period (i.e., with a mean deviation ≥ 0%). The order of the diagnosis entities in each panel is sorted by the value of their post-hiatus mean deviation, in descending order. Rows are colored by the diagnosis category that each entity belongs to. *Abbreviations*: Blind = blindness and vision defects; Neuro = neuro-ophthalmology; Oculoplastics = oculofacial plastics and orbital conditions; OGI = ocular globe injury/intraocular foreign body; Strab = strabismus; RMSPE = root mean squared percentage error; CI =

confidence interval; IOFB = intraocular foreign body; unspec = unspecified; degen = degeneration; PRAO = partial retinal arterial occlusion; DR = diabetic retinopathy; DME = diabetic macular edema; ROP = retinopathy of prematurity; TRAO = transient retinal artery occlusion.

Supplementary Table 4. Diagnosis entities exhibiting partial or sustained recovery in the post-hiatus period.

Entity Name	Post–hiatus Mean Deviation	95% CI	p–value	Recovery Month	Recovery Type	RMSPE
unspec DR w/ DME	0.459	(0.373,0.552)	< 0.001	Jun 2020	Full	0.102
cataract, infantile/juvenile	0.167	(0.122,0.213)	< 0.001	Jun 2020	Full	0.114
corrosion, cornea/conjunctival sac	0.142	(0.082,0.205)	< 0.001	Jun 2020	Full	0.115
other eyelid function disorders	0.125	(0.065,0.189)	< 0.001	Jun 2020	Full	0.102
ROP, stage 3	0.118	(0.064,0.175)	< 0.001	Jun 2020	Full	0.082
ocular laceration w/o prolapse	0.088	(0.037,0.141)	< 0.001	Jun 2020	Full	0.120
optic chiasm disorders	0.049	(0.017,0.082)	0.00144	Jun 2020	Partial	0.116
ROP, stage unspec	0.047	(–0.004,0.101)	0.06543	Jun 2020	Full	0.108
unspec DR w/o DME	0.043	(–0.005,0.094)	0.06818	Jun 2020	Full	0.108
ROP, stage 2	0.042	(–0.019,0.108)	0.17133	Jun 2020	Full	0.080
other corneal deformities	0.018	(–0.011,0.047)	0.21815	Nov 2020	Full	0.064
TRAO	0.014	(–0.015,0.045)	0.33402	Jun 2020	Partial	0.087
iridodialysis	0.013	(–0.016,0.042)	0.37551	Jun 2020	Full	0.084
keratoconjunctivitis – other/unspec	0.012	(–0.001,0.026)	0.0619	Mar 2021	Full	0.108
retrolental fibroplasia	0.004	(–0.047,0.059)	0.87671	Jun 2020	Partial	0.093
eyelid/periocular superficial injury	–0.206	(–0.24,–0.172)	< 0.001	Nov 2020	Partial	0.072
sympathetic uveitis	–0.191	(–0.228,–0.152)	< 0.001	Mar 2021	Partial	0.087
pupillary function anomalies	–0.190	(–0.218,–0.161)	< 0.001	Mar 2021	Partial	0.076
other orbital disorders	–0.186	(–0.212,–0.158)	< 0.001	Feb 2021	Partial	0.083
other binocular eye mvmt dis	–0.182	(–0.216,–0.147)	< 0.001	Mar 2021	Partial	0.100
orbit inflammation – acute	–0.180	(–0.217,–0.143)	< 0.001	Jun 2020	Partial	0.092
lens fragments post–cataract surg	–0.179	(–0.215,–0.14)	< 0.001	Mar 2021	Partial	0.091
other spec visual disturbances	–0.176	(–0.216,–0.135)	< 0.001	Sep 2020	Full	0.103
hyphema	–0.165	(–0.199,–0.13)	< 0.001	Sep 2020	Full	0.090
anterior uveitis – acute, other/unspec	–0.162	(–0.189,–0.134)	< 0.001	Dec 2020	Partial	0.107
eyelid edema	–0.159	(–0.2,–0.116)	< 0.001	Jun 2020	Full	0.106
ocular pain	–0.157	(–0.186,–0.126)	< 0.001	Jun 2021	Partial	0.093
papilledema	–0.154	(–0.182,–0.124)	< 0.001	Feb 2021	Partial	0.078
zoster ocular disease	–0.152	(–0.192,–0.111)	< 0.001	Jun 2020	Full	0.120
FB, conjunctival sac	–0.151	(–0.183,–0.118)	< 0.001	Feb 2021	Partial	0.067
choroidal rupture	–0.148	(–0.179,–0.116)	< 0.001	Dec 2020	Partial	0.121
benign neoplasm – unspec	–0.144	(–0.168,–0.119)	< 0.001	Feb 2021	Partial	0.091
corneal ulcer – perforated	–0.142	(–0.169,–0.113)	< 0.001	Jun 2020	Partial	0.084
xanthelasma	–0.141	(–0.169,–0.111)	< 0.001	Dec 2020	Partial	0.084
choroidal degenerations	–0.138	(–0.16,–0.115)	< 0.001	Jun 2021	Partial	0.064
heterophoria	–0.135	(–0.167,–0.102)	< 0.001	Dec 2020	Partial	0.094
lens dislocation	–0.133	(–0.153,–0.112)	< 0.001	Jun 2021	Partial	0.056
eyelid cysts	–0.131	(–0.16,–0.102)	< 0.001	Feb 2021	Partial	0.078
anisometropia/aniseikonia	–0.130	(–0.156,–0.104)	< 0.001	Dec 2020	Partial	0.075
eyelid/periocular open wound	–0.129	(–0.171,–0.085)	< 0.001	Jun 2020	Full	0.119
optic nerve/pathway injury	–0.128	(–0.166,–0.087)	< 0.001	Jun 2020	Partial	0.111
burn, corneal/conjunctival sac	–0.126	(–0.165,–0.085)	< 0.001	Sep 2020	Partial	0.103
dacryoadenitis – acute	–0.124	(–0.173,–0.073)	< 0.001	Jun 2020	Full	0.119
corneal ulcer – unperforated, w/ str involv	–0.122	(–0.145,–0.099)	< 0.001	Feb 2021	Partial	0.061
corneal neovascularization	–0.122	(–0.141,–0.102)	< 0.001	Feb 2021	Partial	0.067
other scleral disorders	–0.120	(–0.142,–0.097)	< 0.001	Feb 2021	Partial	0.070
eyelid retraction	–0.119	(–0.152,–0.085)	< 0.001	Sep 2020	Full	0.066
interstitial/deep keratitis	–0.116	(–0.131,–0.102)	< 0.001	Feb 2021	Partial	0.054
glaucoma, PACG – other/unspec	–0.116	(–0.144,–0.087)	< 0.001	Feb 2021	Full	0.074
other hereditary corneal dystrophies	–0.115	(–0.132,–0.097)	< 0.001	Jun 2021	Partial	0.048
central serous chorioretinopathy	–0.115	(–0.131,–0.098)	< 0.001	Jun 2021	Partial	0.056
corneal membrane changes	–0.113	(–0.142,–0.083)	< 0.001	Aug 2021	Full	0.083
wet AMD, inactive scar	–0.112	(–0.137,–0.086)	< 0.001	Jun 2021	Partial	0.054
intermittent heterotropia	–0.112	(–0.146,–0.076)	< 0.001	Sep 2020	Full	0.082
conjunctival scars	–0.112	(–0.142,–0.08)	< 0.001	Feb 2021	Partial	0.099
keratitis – infectious	–0.110	(–0.138,–0.082)	< 0.001	Nov 2020	Partial	0.064
dry AMD, adv stage w/ subfov involv	–0.107	(–0.133,–0.08)	< 0.001	Mar 2021	Partial	0.063
glaucoma, PACG – chronic	–0.105	(–0.122,–0.088)	< 0.001	Jun 2021	Partial	0.046
diplopia	–0.104	(–0.128,–0.079)	< 0.001	Mar 2021	Partial	0.052
BRAO	–0.104	(–0.131,–0.077)	< 0.001	Jun 2021	Partial	0.075
secondary uveitis – infectious	–0.103	(–0.131,–0.074)	< 0.001	Jun 2020	Full	0.072
other endophthalmitis	–0.102	(–0.124,–0.08)	< 0.001	Sep 2020	Partial	0.070
lagophthalmos	–0.102	(–0.123,–0.079)	< 0.001	Dec 2020	Partial	0.072
episcleritis	–0.102	(–0.126,–0.077)	< 0.001	Sep 2020	Partial	0.055
ocular laceration w/ prolapse	–0.101	(–0.135,–0.066)	< 0.001	Jun 2020	Full	0.100
cystoid macular degeneration	–0.101	(–0.117,–0.084)	< 0.001	Jun 2021	Partial	0.045
vitreous prolapse	–0.099	(–0.133,–0.064)	< 0.001	Jun 2020	Full	0.093
other refraction/accommodation disorders	–0.099	(–0.126,–0.071)	< 0.001	Feb 2021	Partial	0.062
chorioretinal scars following detach surg	–0.092	(–0.112,–0.072)	< 0.001	Jun 2021	Partial	0.075
other postop complications	–0.091	(–0.117,–0.064)	< 0.001	Oct 2020	Partial	0.122
other subj visual disturb	–0.090	(–0.118,–0.061)	< 0.001	Dec 2020	Full	0.072
benign neoplasm – conjunctiva	–0.090	(–0.117,–0.061)	< 0.001	Sep 2020	Full	0.076
esotropia	–0.087	(–0.11,–0.063)	< 0.001	Dec 2020	Partial	0.060
anterior uveitis – acute, recurrent	–0.086	(–0.118,–0.053)	< 0.001	Sep 2020	Full	0.081
transient visual loss	–0.082	(–0.109,–0.053)	< 0.001	Jun 2020	Full	0.081
amblyopia, unspec	–0.081	(–0.104,–0.058)	< 0.001	Dec 2020	Partial	0.050

Diagnosis Category

- Blind
- Cataract
- Cornea/External
- Glaucoma
- Neuro
- Oculoplastics
- OGI
- Other
- PO
- Refra
- Retina/Vitreous
- Strab
- Uveitis

(Continued on the next page)

panuveitis	−0.080	(−0.099,−0.06)	< 0.001	Jun 2021	Partial	0.041
malignant neoplasm – conjunctiva	−0.080	(−0.108,−0.052)	< 0.001	Nov 2020	Partial	0.078
glaucoma, secondary to eye infl	−0.079	(−0.097,−0.06)	< 0.001	Feb 2021	Partial	0.049
FB, cornea	−0.078	(−0.115,−0.039)	< 0.001	Jun 2020	Partial	0.085
scleritis	−0.074	(−0.089,−0.057)	< 0.001	Jun 2021	Partial	0.043
PDR, stable	−0.074	(−0.101,−0.045)	< 0.001	Jun 2020	Partial	0.075
PDR w/o DME	−0.073	(−0.094,−0.052)	< 0.001	Mar 2021	Partial	0.051
recession of chamber angle	−0.072	(−0.094,−0.05)	< 0.001	Dec 2020	Partial	0.063
oculomotor nerve palsy	−0.071	(−0.119,−0.022)	0.0087	Jun 2020	Full	0.107
RPE detachment, hemorrhagic	−0.070	(−0.098,−0.042)	< 0.001	Jun 2020	Full	0.105
angoid streaks of macula	−0.070	(−0.09,−0.049)	< 0.001	Jun 2020	Partial	0.049
dacryoadenitis – chronic	−0.069	(−0.11,−0.026)	0.00202	Sep 2020	Full	0.099
abducent nerve palsy	−0.069	(−0.097,−0.041)	< 0.001	Jun 2020	Full	0.057
anterior uveitis – acute, primary	−0.067	(−0.098,−0.035)	< 0.001	Jun 2020	Full	0.082
malignant neoplasm – retina	−0.063	(−0.11,−0.012)	0.0146	Aug 2020	Partial	0.090
keratoconjunctivitis – dry eye syndrome	−0.062	(−0.087,−0.036)	< 0.001	Sep 2020	Full	0.094
anterior uveitis – other	−0.062	(−0.092,−0.03)	< 0.001	Sep 2020	Partial	0.082
glaucoma, absolute	−0.056	(−0.077,−0.034)	< 0.001	Dec 2020	Full	0.050
optic neuritis	−0.055	(−0.089,−0.02)	0.00372	Jun 2020	Full	0.071
anterior uveitis – chronic	−0.055	(−0.081,−0.029)	< 0.001	Jun 2020	Full	0.059
CRAO	−0.054	(−0.08,−0.028)	< 0.001	Jun 2020	Full	0.075
glaucoma, PACG – acute	−0.051	(−0.08,−0.02)	0.00173	Sep 2020	Full	0.064
ROP, stage 1	−0.048	(−0.081,−0.014)	0.00699	Jun 2020	Full	0.096
choroidal hemorrhage	−0.044	(−0.068,−0.019)	< 0.001	Dec 2020	Full	0.080
benign neoplasm – orbit	−0.042	(−0.074,−0.008)	0.01394	Nov 2020	Partial	0.099
unspec macular degeneration	−0.041	(−0.074,−0.007)	0.01867	Dec 2020	Full	0.104
enophthalmos	−0.039	(−0.077,0.001)	0.05857	Jun 2020	Full	0.111
RD, traction	−0.037	(−0.051,−0.023)	< 0.001	Jun 2021	Partial	0.040
aphakia	−0.033	(−0.057,−0.009)	0.00857	Sep 2020	Full	0.052
corneal degen – other/unspec	−0.032	(−0.073,0.01)	0.13412	Sep 2020	Partial	0.081
cataract, traumatic	−0.031	(−0.06,−0.001)	0.04269	Jun 2020	Partial	0.069
herpesviral ocular disease	−0.030	(−0.05,−0.009)	0.00486	Jun 2020	Partial	0.070
posterior uveitis	−0.027	(−0.041,−0.012)	< 0.001	Dec 2020	Partial	0.044
hereditary choroidal dystrophies	−0.026	(−0.07,0.021)	0.26877	Jun 2020	Partial	0.099
orbit penetrating wound w/ FB	−0.025	(−0.072,0.023)	0.29921	Jun 2020	Full	0.119
retinal vasculitis	−0.018	(−0.035,−0.001)	0.04106	Jun 2021	Partial	0.061
IOL displacement	−0.018	(−0.063,0.029)	0.44564	Sep 2020	Full	0.091
blebitis	−0.017	(−0.07,0.041)	0.5559	Jun 2020	Full	0.088
retinal neovascularization	−0.013	(−0.043,0.018)	0.40523	Jun 2020	Full	0.069
retained foreign body – eyelid	−0.010	(−0.039,0.02)	0.49789	Sep 2020	Full	0.113

Diagnosis entities that met definitions of partial or sustained recovery in the post-hiatus period. The order of the diagnosis entities in each panel is sorted by the value of their post-hiatus mean deviation, in descending order. Rows are colored by the diagnosis category that each entity belongs to. *Abbreviations*: Blind = blindness and vision defects; Neuro = neuro-ophthalmology; Oculoplastics = oculofacial plastics and orbital conditions; OGI = ocular globe injury/intraocular foreign body; Strab = strabismus; Refra = refractive error; RMSPE = root mean squared percentage error; CI = confidence interval; DR = diabetic retinopathy; DME = diabetic macular edema; ROP = retinopathy of prematurity; TRAO = transient retinal artery occlusion; unspec = unspecified; spec = specified; PACG = primary angle closure glaucoma; AMD = age-related macular degeneration; BRAO = branch retinal artery occlusion; FB = foreign body; PDR = proliferative diabetic retinopathy; RPE = retinal pigment epithelium; CRAO = central retinal artery occlusion; IOL = intraocular lens; RD = retinal detachment; mvmt dis = movement disorder; surg = surgery; str involv = stromal involvement; adv stage = advanced stage; subfov involv = subfoveal involvement; detach = detachment; subj = subjective; infl = inflammation.

Supplementary References.

1. Agresti A. *Foundations of Linear and Generalized Linear Models*. John Wiley & Sons; 2015.

2. Ver Hoef JM, Boveng PL. Quasi-Poisson vs. negative binomial regression: how should we model overdispersed count data? *Ecology*. 2007;88(11):2766-2772.

3. Kleiber C, Zeileis A. Applied Econometrics with R. Published online 2008. https://CRAN.R-project.org/package=AER

4. Bourges JL, Boutron I, Monnet D, Brézin AP. Consensus on Severity for Ocular Emergency: The BAsic SEverity Score for Common OculaR Emergencies [BaSe SCOrE]. *J Ophthalmol*. 2015;2015:576983.
