# Supplementary material for: Shifts in ophthalmic care utilization during the COVID-19 pandemic in the US
Source: Commun Med (Lond). 2023 Dec 14;3:181. doi: 10.1038/s43856-023-00416-4 (PMC10721809; doi:10.1038/s43856-023-00416-4)
Supplement: Supplementary file 7 — Description of Additional Supplementary Files [file 43856_2023_416_MOESM7_ESM.pdf]

Description of Additional Supplementary Files

File Name: Supplementary Data 1
Description: Inventory and ICD-10 crosswalk of all 336 diagnosis entities considered for inclusion

File Name: Supplementary Data 2
Description: Counterfactual model performance metrics and other summary statistics for all 336 diagnosis entities considered for inclusion

File Name: Supplementary Data 3
Description: Point estimates, p-values (unadjusted and adjusted), and 95% confidence intervals of monthly deviations for all 261 diagnosis entities included for analysis

File Name: Supplementary Data 4
Description: Source data for the main figures in this paper
