# Supplementary material for: Shifts in ophthalmic care utilization during the COVID-19 pandemic in the US
Source: Commun Med (Lond). 2023 Dec 14;3:181. doi: 10.1038/s43856-023-00416-4 (PMC10721809; doi:10.1038/s43856-023-00416-4)
Supplement: Supplementary file 8 — Reporting Summary [file 43856_2023_416_MOESM8_ESM.pdf]

nature portfolio

Corresponding author(s): Charles Li
Last updated by author(s): Nov 22, 2023

Reporting Summary

Nature Portfolio wishes to improve the reproducibility of the work that we publish. This form provides structure for consistency and transparency in reporting. For further information on Nature Portfolio policies, see our Editorial Policies and the Editorial Policy Checklist.

Statistics

For all statistical analyses, confirm that the following items are present in the figure legend, table legend, main text, or Methods section.

n/a	Confirmed	
☐	☒	The exact sample size (n) for each experimental group/condition, given as a discrete number and unit of measurement
☐	☒	A statement on whether measurements were taken from distinct samples or whether the same sample was measured repeatedly
☐	☒	The statistical test(s) used AND whether they are one- or two-sided<br>*Only common tests should be described solely by name; describe more complex techniques in the Methods section.*
☐	☒	A description of all covariates tested
☐	☒	A description of any assumptions or corrections, such as tests of normality and adjustment for multiple comparisons
☐	☒	A full description of the statistical parameters including central tendency (e.g. means) or other basic estimates (e.g. regression coefficient) AND variation (e.g. standard deviation) or associated estimates of uncertainty (e.g. confidence intervals)
☐	☒	For null hypothesis testing, the test statistic (e.g. F, t, r) with confidence intervals, effect sizes, degrees of freedom and P value noted<br>*Give P values as exact values whenever suitable.*
☒	☐	For Bayesian analysis, information on the choice of priors and Markov chain Monte Carlo settings
☒	☐	For hierarchical and complex designs, identification of the appropriate level for tests and full reporting of outcomes
☐	☒	Estimates of effect sizes (e.g. Cohen's d, Pearson's r), indicating how they were calculated

Our web collection on statistics for biologists contains articles on many of the points above.

Software and code

Policy information about availability of computer code

Data collection	De-identified patient-level electronic health records were extracted from the cloud data warehouse (Amazon Redshift version 1.0.38698) of the American Academy of Ophthalmology IRIS® (Intelligent Research in Sight) Registry. Data extraction, wrangling, and processing was performed with PostgreSQL 8.0.2 to produce monthly time series data (from January 2017 to December 2021) for each diagnosis entity included in this study, and in accordance with a set of common inclusion/exclusion criteria applied across all diagnosis entities.
Data analysis	All statistical analyses and modeling (including the training and selection of predictive models of counterfactual utilization levels) and data visualizations were conducted with R (version 4.1.0) in RStudio (version 1.4.1106), using the ggplot2 (version 3.3.6), circlize (version 0.4.16), ComplexHeatmap (version 2.10.0), ggrepel (version 0.9.1), and ggpubr (version 0.4.0) packages. Code for this study is publicly available on Github at https://github.com/charlesli37/covid-oph-dx-utils.

For manuscripts utilizing custom algorithms or software that are central to the research but not yet described in published literature, software must be made available to editors and reviewers. We strongly encourage code deposition in a community repository (e.g. GitHub). See the Nature Portfolio guidelines for submitting code & software for further information.

Data

Policy information about availability of data

All manuscripts must include a data availability statement. This statement should provide the following information, where applicable:

- Accession codes, unique identifiers, or web links for publicly available datasets
- A description of any restrictions on data availability
- For clinical datasets or third party data, please ensure that the statement adheres to our policy

The IRIS® Registry is not a publicly available dataset. However, the minimum data necessary to interpret, verify, and extend the research are currently available in the Supplementary Information and is publicly available on Github (in CSV and PDF formats) at https://github.com/charlesli37/covid-oph-dx-utils. These data include information on the set of ICD-10-CM codes used to define each candidate diagnosis entity (Supplementary Data 1); characteristics of the counterfactual model selected for each candidate diagnosis entity (e.g., model specification, model performance metrics) and other summary statistics for the diagnosis entity (e.g., its average monthly volume) (Supplementary Data 2); and estimated monthly deviations from expectation during the pandemic study period, along with associated unadjusted and adjusted p-values and 95% CIs, for the final set of 261 diagnosis entities selected for study inclusion (Supplementary Data 3).

Research involving human participants, their data, or biological material

Policy information about studies with human participants or human data. See also policy information about sex, gender (identity/presentation), and sexual orientation and race, ethnicity and racism.

Reporting on sex and gender	No information on patient sex or gender were collected for this study.
Reporting on race, ethnicity, or other socially relevant groupings	No information on patient race/ethnicity, or other socially relevant groupings, were collected for this study.
Population characteristics	No patient-level demographic information was collected for this healthcare utilization study. All patients included in the monthly count for a diagnosis entity were documented with an ICD-10-CM code corresponding to that diagnosis at any time during the month, as well as a physician encounter (defined as a procedure, ocular examination, or visit record) on the same date as the ICD-10-CM code documentation.
Recruitment	Patients were not recruited for this retrospective observational study. No patient-level inclusion or exclusion criteria, aside from those already described (See Above), were applied.
Ethics oversight	This study was approved and conducted by the Quality and Data Science Division of the American Academy of Ophthalmology.

Note that full information on the approval of the study protocol must also be provided in the manuscript.

Field-specific reporting

Please select the one below that is the best fit for your research. If you are not sure, read the appropriate sections before making your selection.

☒ Life sciences ☐ Behavioural & social sciences ☐ Ecological, evolutionary & environmental sciences

For a reference copy of the document with all sections, see nature.com/documents/nr-reporting-summary-flat.pdf

Life sciences study design

All studies must disclose on these points even when the disclosure is negative.

Sample size	Sample sizes were not predetermined. Since the IRIS® Registry is the United States' first comprehensive eye disease registry and is one of the largest single-specialty clinical databases in the world, the electronic health records queried for each diagnosis entity were collected at a scale that is generally comparable to, or larger than, those of existing studies. Candidate diagnosis entities with particularly small sample sizes (e.g., single-digit monthly patient counts) were excluded from analysis. The scale of the sample size for each diagnosis entity (i.e., average monthly volume of patients observed) is reported in Supplementary Data 2.
Data exclusions	All exclusion criteria were pre-established. To ensure that the monthly counts of patients observed with each diagnosis reflected true clinical visits (i.e., utilization of healthcare services), the only patient-level exclusion criterion was established as patients who did not have a physician encounter (defined as a procedure, ocular examination, or visit record) documented on the same date as their diagnosis documentation. Furthermore, diagnosis entities with a poor out-of-sample model performance (defined as ≥ 12.5% root-mean-squared percentage error) were excluded from analysis to mitigate potentially unreliable conclusions from the data. Finally, we also excluded data from practices that did not contiguously contribute data for all months of the study period to the IRIS® Registry.
Replication	We successfully replicated our findings across different model performance thresholds and sets of inclusion/exclusion criteria (See Above). Specifically, to reduce concerns regarding our choice of a maximum allowable root-mean-squared percentage error, or the criteria we applied to identify patients eligible for inclusion, we conducted sensitivity analyses to demonstrate no major differences in primary outcomes across these different configurations (Supplementary Note 3).

Randomization	Patient-level randomization is not applicable to this study because there were no experimental groups defined within each diagnosis entity. To select a model of counterfactual pandemic utilization among a series of candidate models, leave-out-one-year cross validation was performed on the training data (2017-2019) to identify the model with the best predictive ability for each diagnosis entity.
Blinding	Blinding is not applicable to this retrospective observational study because this study did not include an intervention to ongoing clinical practice.

Reporting for specific materials, systems and methods

We require information from authors about some types of materials, experimental systems and methods used in many studies. Here, indicate whether each material, system or method listed is relevant to your study. If you are not sure if a list item applies to your research, read the appropriate section before selecting a response.

Materials & experimental systems

n/a	Involved in the study
☒	☐ Antibodies
☒	☐ Eukaryotic cell lines
☒	☐ Palaeontology and archaeology
☒	☐ Animals and other organisms
☒	☐ Clinical data
☒	☐ Dual use research of concern
☒	☐ Plants

Methods

n/a	Involved in the study
☒	☐ ChIP-seq
☒	☐ Flow cytometry
☒	☐ MRI-based neuroimaging
